# *Ndrg3* gene regulates DSB repair during meiosis through modulation the ERK signal pathway in the male germ cells

**DOI:** 10.1038/srep44440

**Published:** 2017-03-14

**Authors:** Hongjie Pan, Xuan Zhang, Hanwei Jiang, Xiaohua Jiang, Liu Wang, Qi Qi, Yuan Bi, Jian Wang, Qinghua Shi, Runsheng Li

**Affiliations:** 1 WHO Collaborating Center for Research in Human Reproduction, Key Laboratory of Contraceptive Drugs and Devices of NPFPC, Shanghai Institute of Planned Parenthood Research, Shanghai, 200032, China; 2 Institute of Reproduction and Development, Fudan University, Shanghai, 200032, China; 3 Laboratory of Molecular and Cell Genetics, CAS Key Laboratory of Innate Immunity and Chronic Disease, CAS Hefei Institutes of Physical Science, Hefei National Laboratory for Physical Sciences at Microscale, School of Life Sciences, University of Science & Technology of China, Hefei, 230027, China

## Abstract

The N-myc downstream regulated gene (NDRG) family consists of 4 members, NDRG-1, -2, -3, -4. Physiologically, we found *Ndrg3*, a critical gene which led to homologous lethality in the early embryo development, regulated the male meiosis in mouse. The expression of *Ndrg3* was enhanced specifically in germ cells, and reached its peak level in the pachytene stage spermatocyte. Haplo-insufficiency of *Ndrg3* gene led to sub-infertility during the male early maturation. In the *Ndrg3*^+/−^ germ cells, some meiosis events such as DSB repair and synaptonemal complex formation were impaired. Disturbances on meiotic prophase progression and spermatogenesis were observed. In mechanism, the attenuation of pERK1/2 signaling was detected in the heterozygous testis. With our primary spermatocyte culture system, we found that lactate promoted DSB repair via ERK1/2 signaling in the male mouse germ cells *in vitro*. Deficiency of *Ndrg3* gene attenuated the activation of ERK which further led to the aberrancy of DSB repair in the male germ cells in mouse. Taken together, we reported that *Ndrg3* gene modulated the lactate induced ERK pathway to facilitate DSB repair in male germ cells, which further regulated meiosis and subsequently fertility in male mouse.

Spermatogenesis, by which the spermatogonia develop into highly specialized cells, spermatozoa, is the most essential and complex process in male reproduction^1^. Abnormal alterations of the events such as DSB repair and energy metabolism *et al*. in germ cells are potentially involved in hypo-spermatogenesis or even lead to ultimately male infertility^2^,^3^,^4^.

The programmed induction of the DSBs during meiosis allows the exchange of genetic material between homologous chromosomes which increases the genome diversity^5^,^6^,^7^. However, the DSBs are highly lethal lesions that jeopardize genome integrity^8^. Two major strategies, homologous recombination (HR) and non-homologous end joining (NHEJ) are used to control the DSB repair tightly^9^,^10^,^11^,^12^. DSBs provoke an extensive reaction in neighboring chromatin involving the covalent modifications of histones. Phosphorylation of the histone H2AX on serine 139 of its C-terminal tail (γH2AX) is used as a biomarker for cellular response to DSBs^13^,^14^,^15^. The γH2AX/MDC1 signaling pathway participates into both the HR and NHEJ DNA repair pathways^16^,^17^. Defects in DSB repair causes abnormal recombination and arrest of spermatogenesis, which ultimately result in male infertility^18^.

Testis, a tightly compartmentalized organ, is a place where the male germ cell matures. A metabolic profile shift from aerobic to anaerobic pathways in germ cells takes place as the differentiation of the male germ cells from spermatogonia to spermatozoa^4^. The metabolite lactate secreted from the sertoli cells is utilized as the central fuel for ATP producing during the male meiosis^3^,^4^,^19^,^20^. Latest reports showed that lactate could activate Raf-ERK signaling by binding to NDRG3 to mediate the lactate triggered hypoxia responses^21^,^22^.

*Ndrg3* belongs to the N-myc down-regulated gene (NDRG) family^23^,^24^. Previous reports have suggested the involvement of the NDRG family in the early life development such as organ formation^25^,^26^,^27^,^28^, somite differentiation^29^. The multiple expression of NDRG3 in human tissues and organs, with the highest expression in testis^23^,^30^ is suggesting the presence of some important function of NDRG3 physiologically, especially in testis. The 5α-dihydrotestosterone regulated *Ndrg3* at the beginning of spermatogenesis further implied the function of *Ndrg3* gene may possibly be involved in spermatogenesis^31^. To date, however, little is known about the exact role of NDRG3 involved in such process.

In this study, we detected the temporo-spatial expression pattern of *Ndrg3*. It was tightly associated with meiosis progression in testis. Our genetically modified mouse model showed the haplo-insufficiency of *Ndrg3* gene impaired the meiosis in the male germ cells, indicating the biological role of *Ndrg3* in spermatogenesis. Our approach based on the *in vitro* primary germ cell culture further showed that *Ndrg3* was required for the lactate induced DSB repair via modulating the ERK1/2 pathway. This study will also be helpful for providing a new prospect that how metabolite lactate influences meiosis in the male germ cells.

## Results

### The *Ndrg3* expression and distribution in testis

**Figure 1:**
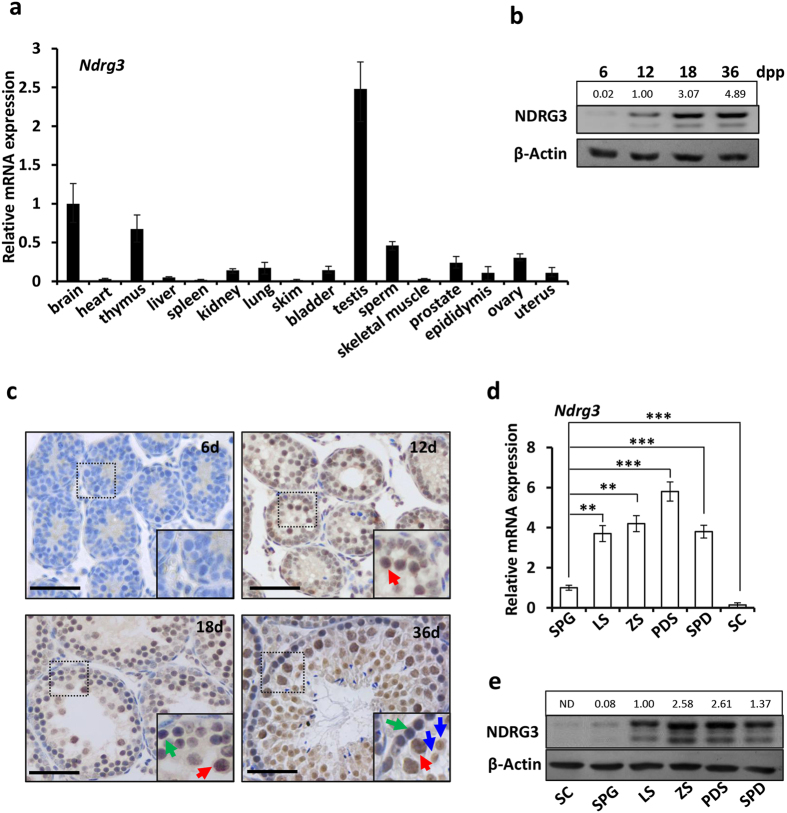
The expression of NDRG3 is highly induced in spermatognia and spermatocytes. (**a**) Relative expression of *Ndrg3* mRNA in mouse tissues was quantified by real-time PCR. (**b**) Western blot assay showed the expression pattern of NDRG3 in testes of mice at the indicated time points. (**c**) Immunohistochemistry showed the spatiotemporal expression pattern of NDRG3 in mouse testes. Note the increase of the protein level of NDRG3 specifically mainly in spermatogonia (green arrow), spermatocytes (red arrow) and spermatids (blue arrow). (**d**) The relative expression of *Ndrg3* mRNA in spermatogonia, spermatocytes of different sub-stages in meiosis prophase I and sertoli cells was determined by real-time PCR. (**e**) Western blot assay showed the expression of NDRG3 in spermatogonia, spermatocytes of different sub-stages in meiosis prophase I and sertoli cells. Scal bar, 25 μm. Abbreviation: dpp, day post-partum; SPG, spermatogonia; LS, leptotene spermatocyte; ZS, zygotene spermatocyte; PDS, pachytene and diplotene spermatocyte; SPD, spermatid; SC, sertoli cell. Full-length blots are presented in the Supplemental Figure 9.

Given the highest expression of human NDRG3 existing in testis, we examined the distribution of *Ndrg3* in mouse tissues by real-time PCR assay^30^. The mRNA level of *Ndrg3* was detected highest in testis compared with other tissues (Fig. 1a). To further investigate the protein level of NDRG3 in testis, we analyzed its expression in the indicated ages. Western blot assay showed that the basal protein level of NDRG3 in testis was very low at 6 days postpartum (dpp), but strikingly induced at 12 dpp and 18 dpp significantly. An even higher expression level of NDRG3 was detected at 36 dpp (Fig. 1b). These data showed that the NDRG3 protein level was raised during the sexual maturation in male mice. To further identify the cell types in which NDRG3 was mainly expressed, the immunohistochemistry assay was carried out. The results showed that NDRG3 protein was detected specifically in germ cells including spermatogonia (green arrows), spermatocytes (red arrows) and spermatids (blue arrows) at 12–36 dpp (Fig. 1c), indicating that the *Ndrg3* gene functioned in the male germ cell development during the spermatogenesis. We further compared the mRNA and protein levels of NDRG3 in spermatogonia, meiosis I prophase subgroups (leptotene, zygotene and pachytene & diplotene stages), spermatids and sertoli cells. Both of the results showed the expression of NDRG3 was exclusively in germ cells and largely induced in the meiosis I prophase spermatocytes and spermatids compared with the spermatogonium group. (Fig. 1d and e), implying that NDRG3 exerted the physiological role shortly after the initiation of the meiosis.

### Establishment of NDRG3 deficient mice

**Figure 2:**
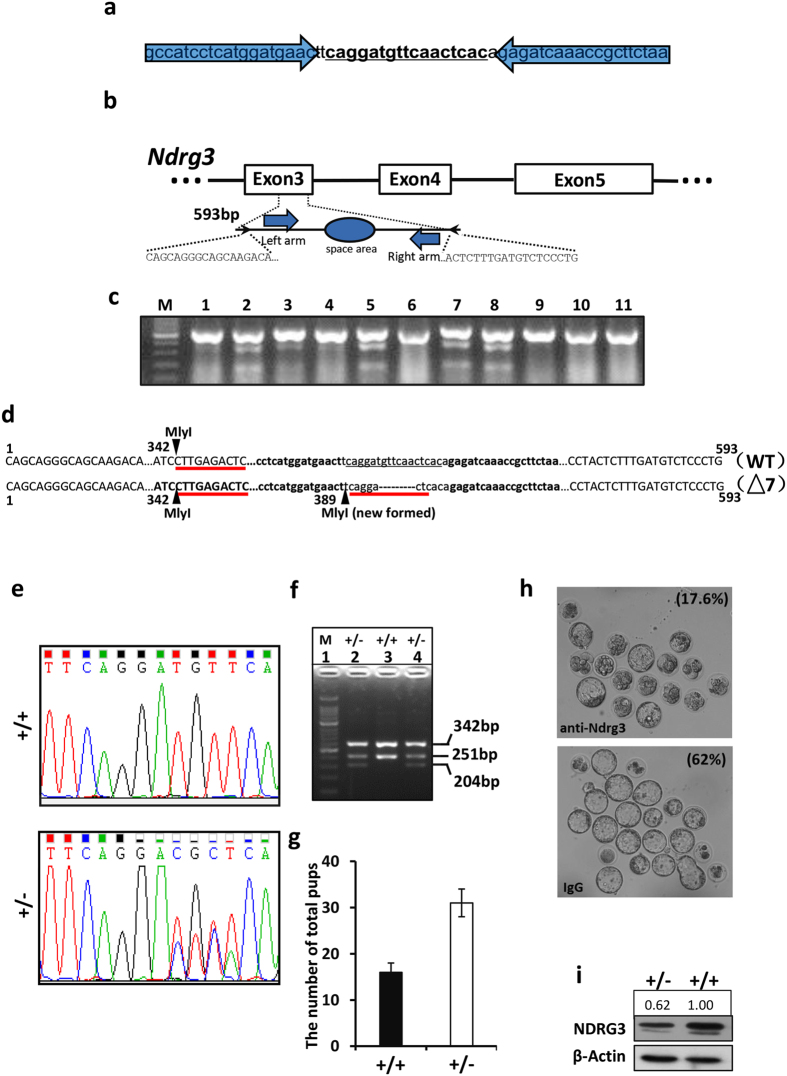
Preparation of *Ndrg3* knockout mouse by TALEN-mediated gene targeting. (**a**) Double-stranded DNA sequence of the *Ndrg3* locus that was targeted with TALENs. The TALEN binding sites were represented by the blue transparent arrows and the spacer region was underlined. (**b**) Diagram of the partial *Ndrg3* gene body showing the TALEN binding sites (blue bold arrows). (**c**) Agarose gel photograph of Surveyor Nuclease Assay demonstrated digestion products of the predicted size from pup #2, #3, #5, #7, #8 and the WT pup #1, #4, #6, #9, #10, #11. (**d**) The DNA sequence of the wild type (WT) sequence of *Ndrg3* with TALEN binding sites in bold, the spacer region underlined, and the MlyI restriction site used for genotyping. Immediately beneath the wild type sequence was the sequence derived from cloned PCR products from founder mouse pup #2 demonstrating that 1 clone was wild type and 1 clone harbored a 7-bp nucleotides deletion (Δ7). (**e**) Sequencing of wild type and the heterozygous alleles. Note a double peak of the signal was detected after the “TTCAGGA” nucleotide. (**f**) Agarose gel photograph of genotyping results after PCR products from wild type (+/+), heterozygous (+/−) mice were digested with MlyI. The wild type allele produced MlyI products of 342 and 251 bp fragments. The heterozygous allele with two MlyI sites produces 342, 251 and 204 bp fragments. (**g**) The mean number of the total litters from the heterozygous mice. (**h**) NDRG3 was required for the early development of the embryo. The one-cell stage embryos were injected with the Neutralization antibody against to NDRG3 (100 ng) and the control IgG and cultured in KSOM medium (Merk Millpore), 37 °C, 5% CO_2_. 4~5 days later, the mean percentage of the neutralized group that developed to the blastocyst stage was 17.6%, whereas the IgG control group was 62%. N = 6. (**i**) Western blot assay showed the level of the NDRG3 protein in the wild type and heterozygous testes of mice. Full-length gel and blots are presented in the Supplemental Figure 10.

To determine the role of *Ndrg3* gene during spermatogenesis, we generated the C57BL/6 mice in which the *Ndrg3* gene was disrupted by TALENs. In short, the TALENs were constructed to target the DNA sequence of the mouse *Ndrg3* gene (Fig. 2a) as illustrated graphically in Fig. 2b. A 593 bp PCR product was amplified from each offspring and subjected to the endonuclease survey (T7E1), which cleaved the PCR amplicon into ~380-bp and ~210-bp fragments. Agarose gel photograph of the surveyor nuclease assay demonstrated the digestion products of predicted size from pup #2, #3, #5, #7, #8 with a ~380-bp and a ~210-bp fragments (Fig. 2c). The above chimeric offspring strains were then subjected to the DNA sequencing and one of the strains (#2) that 7-base deletion in the space area (caggatgttcaactcac) was detected (Fig. 2d). The strain #2 chimeric offspring were crossed to the C57BL/6 background wild type mouse to obtain the heterozygous mice. The heterozygous offspring were further confirmed by sequencing (Fig. 2e). However, we failed to obtain the *Ndrg3*^−/−^ homology (Fig. 2f and g). The number of the pups with indicated genotypes was counted. The mean number of the wild type or heterozygous group was 16 or 31 respectively, indicating the homologous lethality effect of *Ndrg3* (Fig. 2g). We examined the genotype of the embryos from E5.5 to the new born pups, but no homologous mouse was found (data not shown). Thus we thought NDRG3 may influence the early embryo development. We performed the neutralization experiment with an anti-NDRG3 antibody and control IgG in the one-cell stage fertilized eggs. As was shown, about 17.6% eggs developed to the blastocyst stage in the antibody treated group, whereas more than 60% eggs developed to that stage in the IgG treated group (Fig. 2h). The neutralization assay here helped to provide a piece of evidence that NDRG3 protein was required during the early embryo development. Western blot of the whole testis lysates from adult mouse testes showed that the *Ndrg3* gene was disturbed in heterozygous resulted in a down regulated NDRG3 protein level (Fig. 2i).

### Haplo-insufficiency of *Ndrg3* impaired the fertility, testis development and spermatogenesis in male mice

**Figure 3:**
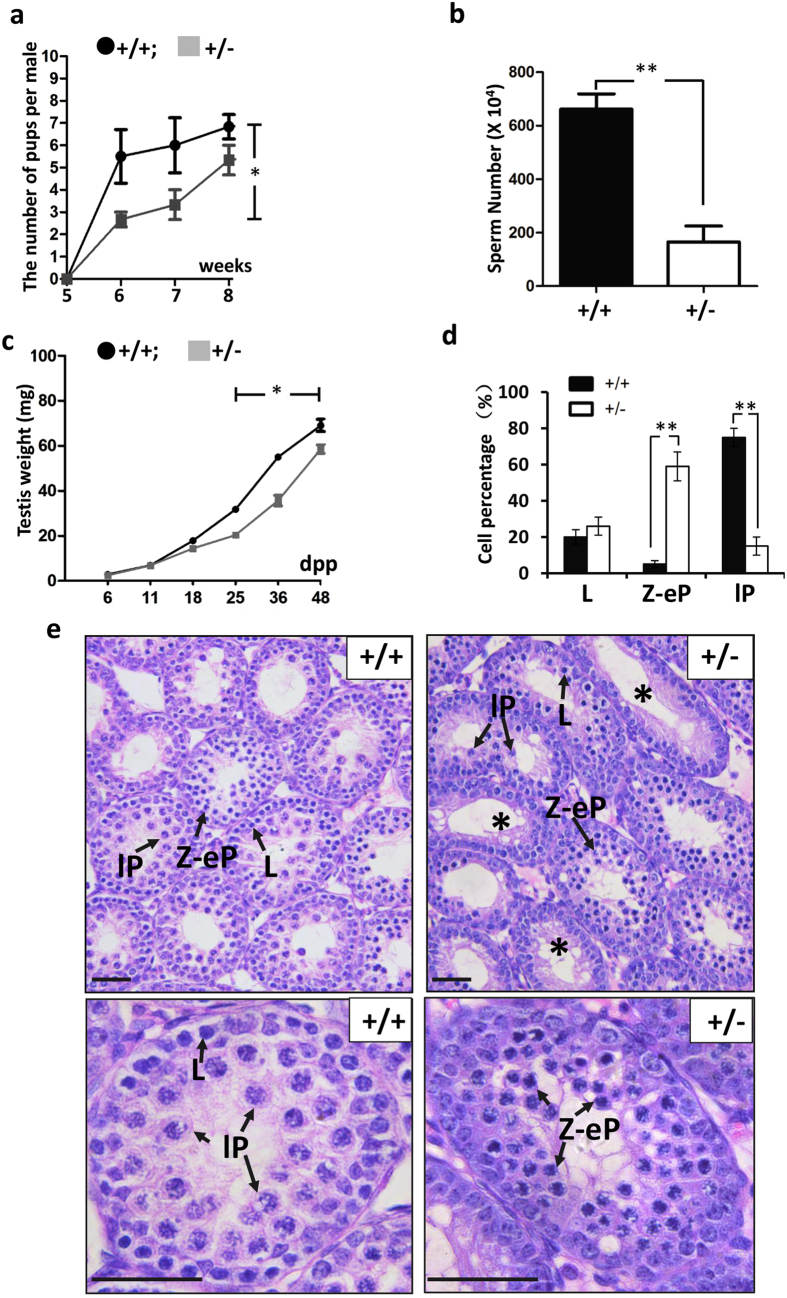
Haplo-insufficiency of *Ndrg3* impairs male reproduction and postnatal testis development. (**a**) The mean number of pups born to mated *Ndrg3*^+/−^ (+/−) and the wild type (+/+). (**b**) The sperm number in epididymides from the 6–8 week wild type and *Ndrg3*^+/−^ mice. (**c**) The mean testis weights of the wild type and *Ndrg3*^+/−^ mice from 6 to 48 dpp. (**d**) The number of the spermatocytes of different sub-stages in meiosis prophase I based on the counting of series sections between the wild type and *Ndrg3*^+/−^ testes. (**e**) H&E staining of the testes from 18 dpp wild type and *Ndrg3*^+/−^ mice. The normal arrangement of the germ cells at 18 dpp was observed and more than half of the total tubules containing pachytene stage spermatocytes in the wild type mouse, while most of the tubules containing the leptotene or zygotene stage like spermatocytes in the *Ndrg3*^+/−^ group. More tubules that the germ cell did not entry into the meiosis were detected in *Ndrg3*^+/−^ testis (star). Scal bar, 25 μm. Abbreviation: L, leptotene; Z, zygotene; eP, early pachytene; lP, late pachytene.

*Ndrg3*^+/−^ male mice were apparently normal in growth (data not shown). To investigate whether the fertility of *Ndrg3*^+/−^ male was impaired, 5- to 8-week-old male heterozygous mice and their wild type littermates were mated with wild type (C57BL6) females (6-week-old) respectively. The number of average pups per *Ndrg3*^+/−^ mouse was significantly decreased, when compared with the wild type litter mates at the indicated time points. The mean number of litters produced by wild type male was about 5.5 at 6-week period whereas the number of *Ndrg3*^+/−^ group was 2.7. The number of the offspring produced by wild type male was 6 whereas the *Ndrg3*^+/−^ group was 3.4 at 7-week period. The wild type group number was about 6.7 while the *Ndrg3*^+/−^ group was 5.2 at 8-week period (Fig. 3a). The number of spermatozoa was significantly lower in *Ndrg3*^+/−^ mice when compared with that in the wild type groups (Fig. 3b) at the time points of 6 to 8 weeks postpartum. The decreased numbers of pups and spermatozoa in the heterozygotes suggested that the deficiency of NDRG3 led to the sub-fertility during the early stage of male maturation. We examined the testis weight of the *Ndrg3*^+/−^ and wild type groups from 6 to 48 dpp. *Ndrg3*^+/−^ and wild type groups showed no apparent difference at 6 and 11 dpp. As the growth continuing, the mean weight number of *Ndrg3*^+/−^ group was16, 20.5, 36 and 58.6 milligram at each time point respectively, which were significantly lower than that observed in the wild type group (Fig. 3c). On the other hand, we did not detect any significant difference in body weight (data not shown) between the two groups. Next, we performed histological analysis between the two group testes at early developmental stages in order to investigate the reasons causing subfertility and reduced testis weight in *Ndrg3*^+/−^ mice. No obvious difference was observed between the wild type and *Ndrg3*^+/−^ testis histology before 8 dpp (data not shown). At 18 dpp, we observed a large number of late pachytene spermatocytes in the wild type tubules whereas the most frequent spermatocytes presented was leptotene or zygotene-like stage cells in the *Ndrg3*^+/−^ testes (Fig. 3d and e). As the *Ndrg3 gene* was high in brain (Fig. 1a), we also determined the testosterone level between the wild type and the *Ndrg3*^+/−^ group, but no significantly changes were found (Supplemental Figure 1), indicating that the testicular tubule defect was mainly due to the development failure of the male germ cells. These above visible defects in spermatocyte development implied the abnormality of meiosis in the *Ndrg3*^+/−^ testes. Taken together, haplo-insufficiency of *Ndrg3* in testis impaired the male fertility, testis maturation, male germ cell development and spermatogenesis.

### Deficiency of *Ndrg3* resulted to the meiosis abnormality

**Figure 4:**
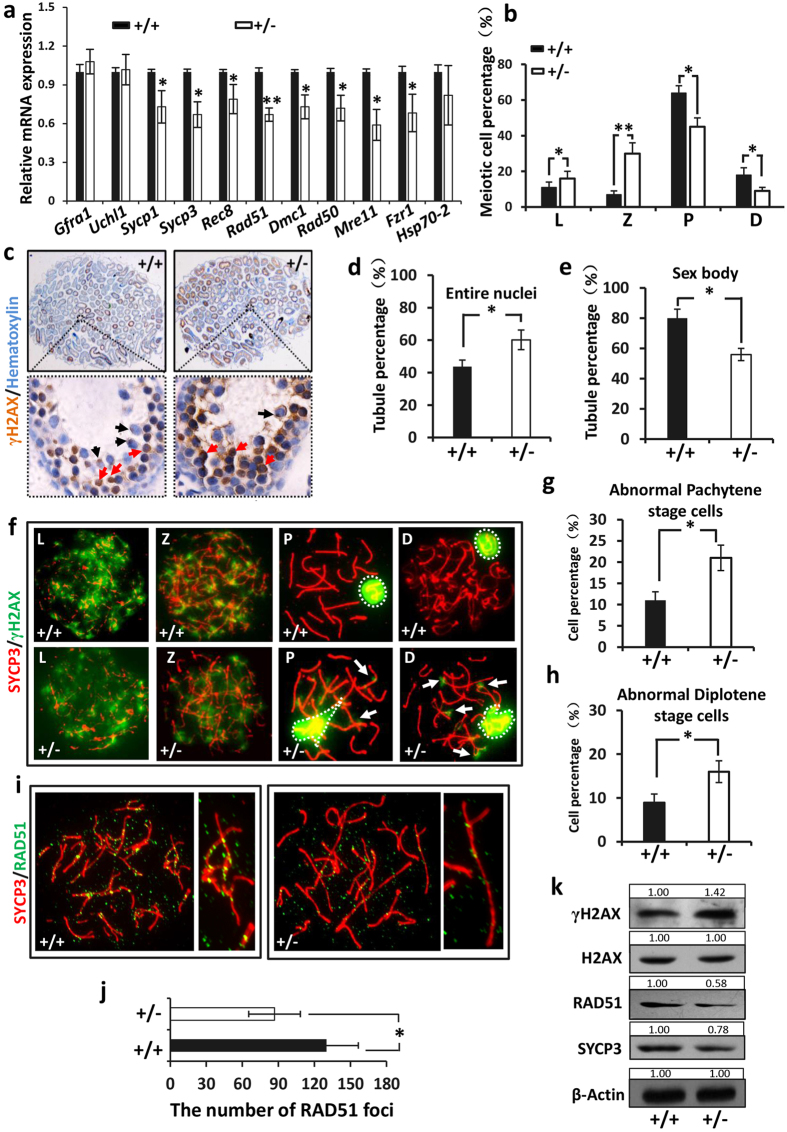
NDRG3 impairment resulted to the meiosis defect. (**a**) The expression of the spermatogonia cell specific (*Uchl1* and *Gfra1*) and meiosis-associated genes (*Sycp1, Sycp3, Rec8, Rad51, Mre11, Rad50, Dmc1* and *Fzr1*) of the 18 dpp *Ndrg3*^+/−^ and wild type testes was examined by real-time PCR analysis. (**b**) Relative amounts of four spermatocyte populations during the prophase I in testes based on analyzing more than 400 spermatocytes each stage. (**c**) IHC assay with γH2AX specific antibody was performed on 18 dpp testes. Punctate positive staining in the sex body region (black arrow). Positive staining throughout the nuclei (red arrow). (**d**) The percentage of the tubules that containing the germ cells with the entire nuclei positive staining. (**e**) The percentage of the tubules that containing the germ cells with the punctate positive staining. (**f**) Double immunofluorescence of surface-spread chromatin preparations of the wild type and *Ndrg3*^+/−^ testes. Synapses of the homologous chromosome were observed by labeling SYCP3 (red), a lateral element of the synaptonemal complex (SC). Initiation and repair of programmed DSB was shown by γH2AX staining (green). Note: At pachytene stage, the γH2AX staining was restricted to the XY chromosomes (white dashed circles) in the wild type spermatocytes, whereas some γH2AX foci remained on asynapsed autosomes (white arrows and dashed regions) indicating the abnormal SCs with the persistence of unrepaired DSB in *Ndrg3*^+/−^. (**g**) The percentage of the pachytene stage spermatocytes with the abnormal synaptonemal complex in the wild type and *Ndrg3*^+/−^ mice. (**h**) The percentage of the diplotene stage spermatocytes with the abnormal synaptonemal complex in the wild type and *Ndrg3*^+/−^ mice. (**i**) Double immunofluorescence of surface-spread chromatin preparations of the wild type and *Ndrg3*^+/−^ testes. SYCP3 (red), RAD51 (green). (**j**) Quantification of RAD51 folci between the wild type and *Ndrg3*^+/−^ zygotene spermatocytes. (**k**) Western blot assay showed the indicated protein levels between the wild type and *Ndrg3*^+/−^ testes. Abbreviation: SC, synaptonemal complex; L, leptotene; Z, zygotene; P, pachytene; D, diplotene. Full-length blots are presented in the Supplemental Figure 11.

According to the location of NDRG3 protein in tubules (Fig. 1) and the physiological defect found in testis (Fig. 3), we hypothesized that haplo-insufficiency of *Ndrg3* affected meiosis in male. Firstly, the spermatogenesis progression analysis was performed using 8 weeks old mice. The morphologies of mid-pachytene spermatocyte, metaphase stage of spermatogonium, metaphase I and metaphase II spermatocyte were distinguished according to the previous study^32^ and the results were shown in Supplemental Figure 2. Based on counting the specific cell number per 1000 spermatocytes, *Ndrg3*^+/−^ mouse resulted in a significant decrease in the frequencies of meiosis I metaphase (MMI) or meiosis II metaphase (MMII) (Table 1), indicating that spermatogenesis was disturbed in *Ndrg3*^+/−^ mice. Secondly, we performed real-time PCR assay to find out which stage of spermatogenesis was affected. As was shown in Fig. 4a, no significant difference in the mRNA levels of *Uchl1* and *Gfra1* was found between the wild type and *Ndrg3*^+/−^ testes (The expression of *Uchl1* and *Gfra1* is high in spermatogonia^33^,^34^), suggesting NDRG3 deficiency did not affect the spermatogonia. However, the mRNA levels of *Sycp3, Sycp1* (synaptonemal complex (SC) proteins establishing the architecture of the meiotic chromosomes), *Rec8* (a cohesion protein that binds sister chromatids together to form the synaptonemal complex^35^), *Rad51, Dmc1, Mre11, Rad50* (DSB repair associated), *Fzr1* (meiosis arrest associated) were found to be down-regulated significantly in the *Ndrg3*^+/−^ testis. These results strongly suggested that the deficiency of *Ndrg3* impaired the events appearing in the early stage of meiosis.

In order to further characterize the function of NDRG3 in the meiotic prophase I progression. Testicular cells were spread and stained with the anti-SYCP3 antibody, and the numbers of the germ cells in the indicated sub-stages of meiotic prophase I were counted, respectively. The relative ratios of the leptotene and zygotene stage cell of *Ndrg3*^+/−^ mice were increased significantly compared with the wild type group (~16% vs ~11%, ~30% vs ~7% respectively), while the relative ratios of pachytene and diplotene stage cells were decreased in *Ndrg3*^+/−^ mice (~45% vs ~64%, ~9% vs ~18% respectively) (Fig. 4b). This indicated that *Ndrg3* deficiency interfered with the transition from zygotene to pachytene in male germ cells.

Dynamic changes in chromatin structure take place along the meiotic process accompanied by the histone H2AX phosphorylation at ser139 (γH2AX) which is triggered by the DNA double strand breaks (DSBs) formation. The phosphorylation of H2AX is detected on large domains of chromatin in the vicinity of the breaks^13^,^14^,^15^. After entering into the meiosis, the γH2AX signal is detected throughout the nuclei from the leptotene stage to the early zygotene stage. As meiosis proceeds to the pachytene stage, γH2AX disappears from synapsed autosomes and is restricted to the unsynapsed sex chromosomes in the sex body^16^. Herein we performed the immunohistochemistry (IHC) assay and our results showed that more than 60.0% tubules containing the germ cells with the entire nuclei stained by γH2AX in the *Ndrg3*^+/−^ group, whereas this staining pattern was detected in 43.7% tubules in the wild type group (Fig. 4c and d). However, about 80% tubules contained the spermatocytes with the punctate positive staining indicating the γH2AX was restricted to the sex body region in the wild type group, whereas the number of these tubules was ~56% in the *Ndrg3*^+/−^ group (Fig. 4c and e). These results suggested that the deficiency of *Ndrg3* impairs DSB repairing in spermatocytes. We further studied the effect of NDRG3 on γH2AX distribution in meiotic male germ cells via spreading assay. We did not detect any significant different staining in the leptotene or early zygotene between the *Ndrg3*^+/−^ and wild type group, suggesting the successful generation of DSB in *Ndrg3*^+/−^ germ cells. At pachytene and diplotene stages, only the sex body (white dashed regions) was stained with γH2AX antibody, as expected, in the wild type spermatocytes, indicating that DSBs disappeared from the autosomes. However, the γH2AX foci remained on the autosomes at pachytene and diplotene stages in the *Ndrg3*^+/−^ spermatocytes, indicating the presence of abnormal DSBs in the pachytene and diplotene in the *Ndrg3* deficient mice. Additionally, some autosomes were also observed in the γH2AX staining sex body region in some pachytene and diplotene spermatocytes in the *Ndrg3*^+/−^ group (Fig. 4f). The number of the spermatocytes at pachytene and diplotene stage with abnormal staining of γH2AX were significantly higher in the *Ndrg3*^+/−^ than in wild type group, respectively (Fig. 4g and h). These results suggested that the deficiency of *Ndrg3* impairs DSB repair in spermatocytes. In zygotene, repair of meiotic DSBs occurs, concomitant with the pairing of homologous chromosomes. Thus an examination of RAD51, a DNA repair marker, was performed between the *Ndrg3*^+/−^ and wild type spermatocytes. A decreased number of the RAD51 foci was observed in *Ndrg3*^+/−^ spermatocytes compared to the wild type group (Fig. 4i and j), strongly suggesting that the haplo-insufficiency of *Ndrg3* led to the impaired DSB repair. The quantification of the indicated protein levels by western blot assay further confirmed the down-regulated DSB repair ability and abnormal DSB retention in the *Ndrg3*^+/−^ testes (Fig. 4k). We also analyzed the homologous chromosomes synapsis (SYCP1) and crossover (MLH1) markers, but no significant difference was detected between the *Ndrg3*^+/−^ and wild type groups (Supplemental Figure 3). Collectively, our results demonstrated that the deficiency of *Ndrg3* gene resulted in the impaired DSB repair ability in zygotene, subsequently resulting in an abnormal presence of DSBs at the stages of pachytene and diplotene in meiosis I which was followed by a disturbed meiosis progression in male mice.

### DSB repair required the lactate induced ERK1/2 signaling

**Figure 5:**
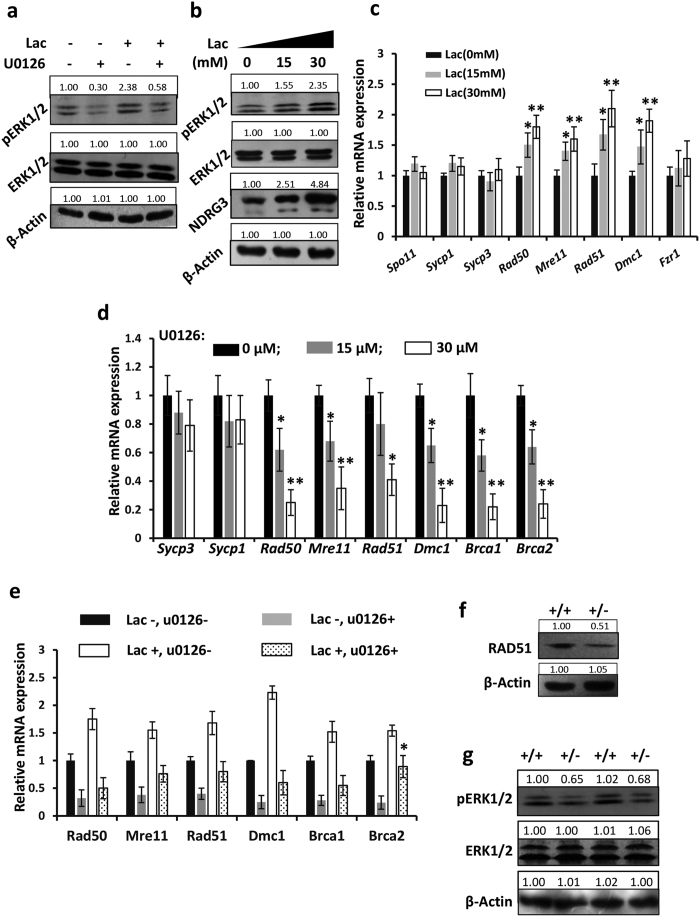
The lactate induced ERK1/2 signaling was required in the DSB repair in the spermatocytes. (**a**) The wild type male germ cells were isolated and cultured with or without lactate (15 mM) or U0126 (15 μM) respectively. The pERK1/2, ERK1/2, and β-Actin levels were determined by western blot assay. (**b**) The pERK1/2, ERK1/2, NDRG3 and β-Actin levels were determined with (15 mM, 30 mM) or without the treatment of lactate (0 mM) in the cultured primary male germ cells. (**c**) The mRNA levels of germ cell specific and the meiosis associated genes were determined in the wild type germ cells after the treatment of lactate *in vitro*. (**d**) Male germ cells were isolated from the wild type testes and cultured with (15 μM and 30 μM) or without (0 μM) U0126. After the treatment of U0126, the mRNA level of *Sycp3, Sycp1, Rad50, Mre11, Rad51, Dmc1, Brca1* and *Brca2* was determined by real-time PCR assay. (**e**) Isolated and cultured male germ cells were treated with or without lactate (30 mM) or U0126 (30 μM) respectively. The mRNA levels of the DSB repair associated genes were determined by real-time PCR assay. (**f**) Western blot assay of the expression of RAD51 in the isolated and cultured wild type and *Ndrg3*^+/−^ male germ cells. (**g**) Western blot assay of the ERK1/2 signal in the wild type and *Ndrg3*^+/−^ testes. Abbreviation: Lac, lactate. Full-length blots are presented in the Supplemental Figure 12.

Lactate, binding with NDRG3, a common product of anaerobic metabolism, not only protects NDRG3 from proteasomal degradation, but also activates the ERK1/2 pathway in the hypoxia responses^21^. Here, we confirmed that the lactate activated the ERK1/2 signaling in male germ cells *in vitro*. As was shown, after added to the cultured primary germ cells, lactate raised the level of pERK1/2 in the wild type male germ cells (Fig. 5a and b). Additionally, Fig. 5b showed that the lactate enhanced the NDRG3 protein level in a dose-depending way, which strongly suggested the inhibitory role of lactate to the NDRG3 proteasomal degradation.

As the MAPK cascade plays complicated roles in meiosis in the male germ cells^36^,^37^,^38^. In order to explore whether the lactate-NDRG3-ERK1/2 axis is required in DSB repair, we measured the expression of some associated genes during meiosis *in vitro*. Our real-time PCR assay showed the mRNA levels of *Rad50, Mre11, Rad51* and *Dmc1* were increased significantly after the treatment with lactate (Fig. 5c), suggesting that lactate participated in the DSB repair via up-regulating the associated genes.

To test whether the DSB repair requires the ERK signal in male germ cells, we isolated the male mouse germ cells and cultured with (15 μM and 30 μM) or without (0 μM) the ERK signal inhibitor U0126. Then real-time PCR assay detected the decreasing of *Rad50, Mre11, Rad51, Dmc1, Brca1* and *Brca2* (Fig. 5d). The down regulation of the above genes suggested the ERK signal was required for DSB repair. The ERK inhibition effect of U0126 was shown by western blot assay (Supplemental Figure 4). The co-effect of U0126 and lactate on DSB repair was analyzed by the real-time PCR assay, a majority of DSB repair associated genes was not induced by lactate in the U0126 treated male germ cells *in vitro* (Fig. 5e), indicating that the lactate induced DSB repair required the activation of ERK1/2 signaling. The protein level of RAD51 was measured in the *Ndrg3*^+/−^ and wild type spermatocytes *in vitro*. The down-regulation of the meiosis associated proteins (Fig. 5f and Supplemental Figure 5) and other DSB repair related genes (Fig. 4a) in *Ndrg3* deficient testes implied that NDRG3 influences the ERK1/2 signaling. We then compared the levels of pERK1/2 between the *Ndrg3*^+/−^ and wild type testes. As was expected, the level of pERK1/2 was lower in the *Ndrg3*^+/−^ than in the wild type groups (Fig. 5g), suggesting an attenuated ERK activation resulted from *Ndrg3* deficiency in mouse testis.

### *Ndrg3* gene modulated the ERK1/2 signaling in the DSB repair

**Figure 6:**
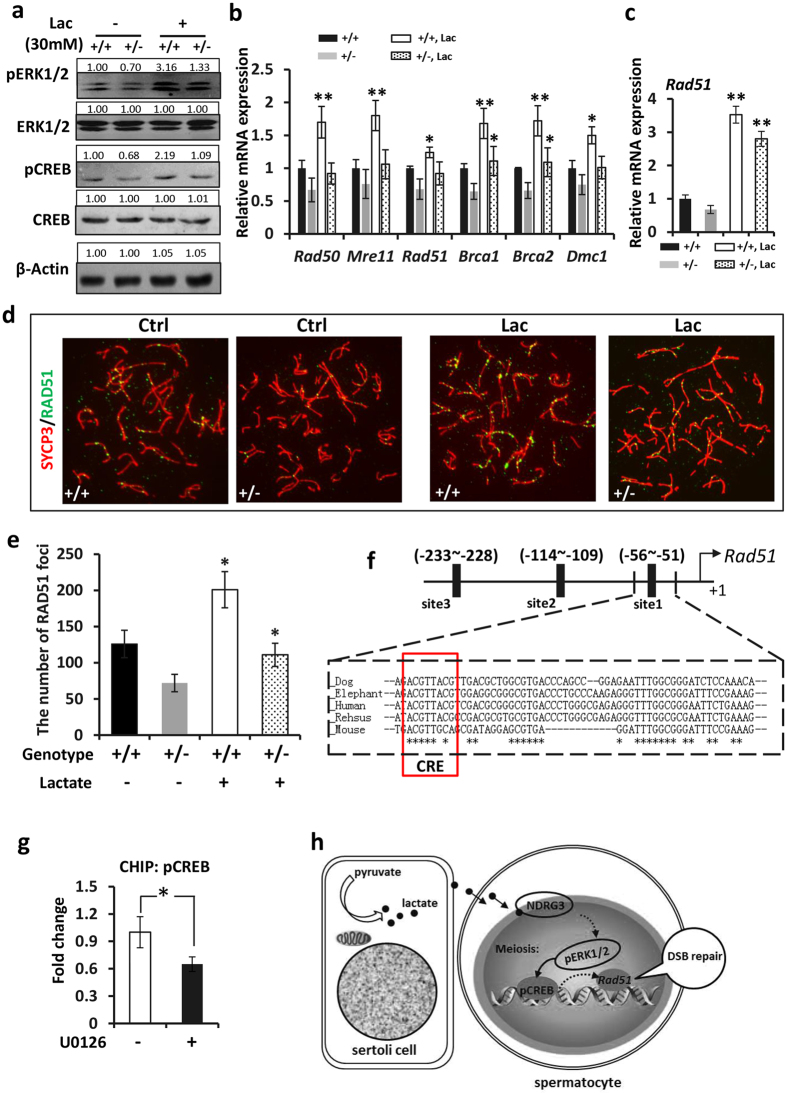
*Ndrg3* gene mediated the ERK1/2 signaling in the DSB repair. (**a**) The wild type and *Ndrg3*^+/−^ male germ cells were isolated and cultured with or without lactate. The pERK1/2, ERK1/2, pCREB, CREB and β-Actin were determined by specific antibodies. (**b**) The mRNA levels of the DSB associated genes were determined by real-time PCR assay. (**c**) The mRNA levels of *Rad51* were determined by real-time PCR assay after 36–38 hrs lactate treatment in wild type and *Ndrg3*^+/−^ male germ cells. (**d**) Surface-spread chromatin preparations of the wild type and *Ndrg3*^+/−^ germ cells. SYCP3 (red), RAD51 (green). (**e**) Quantification of RAD51 folci between the wild type and *Ndrg3*^+/−^ zygotene spermatocytes. (**f**) Diagram of pCREB binding sites (CREs) in the *Rad51* promoter. (**g**) CHIP-qPCR assay of the pCREB/CRE site from −51 to −233 sites. (**h**) Model depicting the molecular mechanisms of NDRG3 mediating Lactate-pERK-pCREB-RAD51 pathway in meiosis DSB repair in the mouse male germ cells. Abbreviation: Lac, lactate. Full-length blots are presented in the Supplemental Figure 13.

We hypothesized that NDRG3 played its meiosis-regulating role in a lactate-dependent way. In the cultured primary testicular cells from 6–8 week-age mice, we found that the pERK1/2 pathway (ERK1/2-pCREB) was enhanced apparently in the wild type germ cells, whereas the pERK1/2 in *Ndrg3*^+/−^ germ cells was up regulated slightly after the treatment of lactate (30 mM) (Fig. 6a), suggesting NDRG3 is required for the lactate-induced activation of ERK1/2.

The mRNA levels of meiosis-associated genes were then examined by the real-time PCR assay. With the treatment of lactate, the DSB repair related genes such as *Rad50, Mre11, Rad51, Dmc1, Brca1* and *Brca2* were increased significantly in the wild type group. But the expressions of these genes were improved in various degrees in *Ndrg3*^+/−^ germ cells (Fig. 6b). Then a longer time treatment of lactate with the spermatocytes were performed between the *Ndrg3*^+/−^ and wild type groups *in vitro* (>36 hours). Some of the DSB repair genes were improved largely both in the wild type and *Ndrg3*^+/−^ spermatocytes (for example, *Rad51*). Figure 6c showed the mRNA level of *Rad51* were increased dramatically both in the *Ndrg3*^+/−^ and wild type spermatocytes significantly. Note 4 fold increase of *Rad51* mRNA detected in the *Ndrg3*^+/−^ spermatocytes with the lactate treatment. Additionally, immunofluorescence assay showed the number of RAD51 foci increased obviously both in wild type and *Ndrg3*^+/−^ spermatocytes, but the number of the foci was still less in the *Ndrg3*^+/−^ group than that in wild type group under the treatment of lactate (Fig. 6d and e). These results gained *in vitro* suggested that insufficiency of NDRG3 impaired the lactate-responsive DSB repair.

To investigate how lactate-ERK-pCREB pathway influences the DSB repair, some DSB repair genes’ promoter regions were analyzed *in silico*. Three putative CRE (pCREB binding site) sequences (TGACGTCA) were found in the promoter region of *Rad51* (−51/−56, −109/−114, −228/−233). One of the three putative CREs (−51/−56) located in a high conserved region (Fig. 6f). Thus we hypothesized that the pCREB binds to the CREs (−51/−233) in the *Rad51* promoter. CHIP-qPCR assay showed U0126 significantly repressed the binding of pCREB to this promoter region approximately by 30% (Fig. 6g). These results demonstrated that the ERK-CREB signaling mediated the DSB repair by regulating the transcript level of *Rad51*.

Taken together, our present data demonstrated that the *Ndrg3* gene regulated the expression of *Rad51*and the DSB repair via modulating the lactate-induced pERK1/2 signal pathway in the mouse male germ cells (Fig. 6h).

## Discussion

Although NDRG3 expresses highest in testis both in human and mouse, its physiological function has not been reported. In this present study, we demonstrated that NDRG3 plays an important role in DSB repair during the meiotic prophase I in testis.

Testis is a naturally oxygen-deprived organ in which lactate is the central energy metabolite for male germ-cell^39^. In testis, lactate also plays other key roles more than just serves as a substrate in metabolism. Previous reports showed that the lactate, which exerted an anti-apoptotic effect on germ cells^40^, was responsible for RNA and protein synthesis and improved spermatogenesis^41^. However, the elucidation of how lactate exerts its role in spermatocytes exactly is still very limited. NDRG3 has been reported to be bound to lactate, and then activate the Raf-ERK pathway in hypoxia^21^. Here, we established the Lactate-NDRG3-ERK1/2-CREB-RAD51 regulatory pathway in male germ cells, in which lactate not only stabilized NDRG3 protein, but also activated ERK signaling in an NDRG3-dependent way. Activation of ERK signaling transcriptionally raised the expression of RAD51 via phosphorylating CREB. Therefore, this pathway represented a new mechanism in DSB repair. Interestingly, our study also revealed a functional link between metabolism and the high expression of NDRG3 in testis. We observed that lactate improved the stability of NDRG3, which provided a piece of evidence for why NDRG3 expression is high in testis.

DSBs are formed by the induction of type II-like topoisomerase SPO11^42^ during the meiotic prophase I. Non-homologous end joining (NHEJ) and homologous recombination (HR) are two major mechanisms applied in DSB repair. MRN complex (consisting of MRE11, RAD50 and NBS1) and a number of kinases (such as ATM, ATR and DNA PKcs) are involved in DNA replication, DNA repair, and signaling to the cell cycle checkpoints^43^. In the *in vitro* assay, we revealed that NDRG3 mediated the DSB related genes via modulating lactate-ERK signaling. However, it should be mentioned that the mRNA levels of the DSB repair related genes (*Rad50, Mre11, Dmc1* and *Rad51*) were partially rescued after the treatment of lactate in the *Ndrg3*^+/−^ group compared with the wild type germ cells indicating that NDRG3 participated more than one signal pathway. The lactate-ERK independent transcription of *Sycp1* and *Sycp3* showed in the *in vitro* assay, together with the down-regulation of the transcript levels of meiosis associated genes (*Sycp1, Sycp3, Fzr1* and *Rec8*) detected *in vivo* further supported this above speculation.

Interestingly, the fertility of male *Ndrg3*^+/−^ mouse was recovered after 10–12 weeks age (Supplemental Figure 6). The phylogenetic analysis of the NDRG gene family demonstrated that the NDRG1 and NDRG3 belonged to one subfamily^44^,^45^. Along with the published data that NDRG1 was up-regulated under hypoxia^46^, therefore, we think NDRG1 might compensate the effect resulted from the deficiency of NDRG3. As a piece of supporting evidence for the hypothesis, we detected that expression of *Ndrg1* was up-regulated after 36 and 48 dpp (Supplemental Figure 7), while the up-regulation of NDRG1 did not exist when NDRG3 mediating the spermatogenesis progression during the first wave.

Numerous studies showed that the activation of ERK signaling either inhibited or promoted the DNA repair according to different cells^38^,^47^,^48^,^49^. An aberrant up-regulation of EGFR signaling in the male germ cells was shown recently to impair the DSB repair with the down regulation of DNA-dependent kinase activation and over-staining of the γH2AX signals, indicating that the hyper-activation of MAPK cascade inhibited the DSB repair in meiosis^38^. In the present study, we showed the requirement of ERK signal pathway during the DSB repair. Inactivation of ERK1/2 cascade impaired the DSB repair with an abnormal retention of the γH2AX signal in male germ cells *in vivo* and decreased a series of DSB repair associated genes. Therefore, we provided a new mouse model showing that hypo-activation of MAPK cascade also affected the DSB repair during meiosis. In a rational manner, it may be speculated that the MAPK cascade is regulated precisely in germ cells, while those factors/chemicals which caused hyper- or hypo-activation of MAPK cascade will eventually result in the failure of DSB repair.

In summary, we have established the Lactate-NDRG3-ERK1/2-CREB-Rad51 regulatory pathway, which plays an important role in the first wave spermatogenesis. However, its significance in male infertility remains unknown and is worth a next approach. As the clinical data have showed a high prevalence of the subfertility was with metabolite disorders^50^, thus a further identification of the NDRG3 interacted proteins and the regulating mechanisms will make a significant contribution to a deeper understanding of male sub-infertility.

## Materials and Methods

### Animals

For fertility testing, 5 to 8 weeks old *Ndrg3*^+/−^ or the wild type males were housed with 6 weeks old wild type C57BL/6 females primed with PMSG (5U) and hCG (5U). The number of the offspring from each pregnant female was counted after birth. All mice were kept under the controlled photoperiod conditions (lights on 07:00–19:00) and supplied with food and sterilized H_2_O *ad libitum*. All experiments were conformed to the regulations drafted by Association for Assessment and Accreditation of Laboratory Animal Care in Shanghai and were approved by the Shanghai Institute of Planned Parenthood Research Center for Animal Research.

### Sperm counting

Epididymides were removed from the wild type and *Ndrg3*^+/−^ mice, incised and incubated in 1 ml buffer containing 75 mM NaCl, 24 mM EDTA, and 0.4% bovine serum albumin (Sigma, A2058) at 34 °C with 5% CO_2_ for 5 minutes to allow sperm to release. The suspension containing sperms was collected and counted with a CASA system (IVOS II Sperm Analyser, Hamilton).

### Hematoxylin and eosin (H&E) staining, immunohistochemistry (IHC)

Testes were collected and immediately fixed in Bouin’s solution for H&E staining and fixed in 4% paraformaldehyde (PFA) for immunohistochemistry. For IHC assay, sections (4–5 μm) were deparaffinized in xylene and rehydrated in gradient alcohols. After antigen retrieval, the blocked sections were incubated with primary antibodies overnight at 4 °C. The sections were incubated with secondary antibodies for 20 minutes the next day and then developed with DAB and counterstained with hematoxylin. Antibodies were diluted as follows: NDRG3, at 1:100 (Santa Cruz Biotechnology), γH2AX (Abcam) at 1:1000. To ensure reproducibility of the results, samples from ≥3 animals were used.

### Meiotic prophase cell spreading and immunofluorescence staining

Spreads of spermatocytes and immunofluorescence staining were prepared according to the previous references^51^,^52^. Briefly, seminiferous tubules were incubated in hypotonic extraction buffer (50 mM Sucrose, 17 mM Sodium citrate, 30 mM Tris (pH 8.2), 2.5 mM DTT, 1 mM PMSF (pH 8.3) and 5 mM EDTA on ice for 20 minutes, minced in 100 mM sucrose, spread on slides and fixed in 1% PFA with 0.1% Triton X-100. Slides were incubated in a humid chamber overnight, dried, and washed in PBS and water containing Photoflo (Kodak, NY, USA). Following blocking in 10% donkey serum and 3% BSA, immunofluorescence staining was performed by incubating with primary antibodies: γH2AX (1:500; abcam) and SYCP3 (1:100; Abcam) overnight at room temperature. Alexa 488 donkey anti-rabbit (1:500, Molecular Probes), Alexa 594 goat anti-mouse (1:200, Molecular Probes) were used as secondary antibodies. Slides were incubated with secondary antibodies at 37 °C for 1 hour in the dark, washed and mounted with Vecta shield cover slips. (Vector Laboratories).

### Primary germ cell preparation

Testicular cells were obtained as previously described^53^. Briefly, the capsules of the testis were removed and the testicular tubules were minced and transferred to a 50 mL Falcon tube. Tissue was suspended in F12/DMEM (Gibco), centrifuged, collected, and then subjected to digestion. Use trypsin/EDTA (0.1 mg/mL; Sigma), DNase (0.02 mg/mL; Sigma), glycine (1 M; Sigma), EDTA (2 mM; Sigma) and STI (0.1 mg/mL; Sigma) to eliminate Leydig cells. Use Collagenase 1 A (0.1 mg/mL; Sigma) and DNase (5 μg/mL; Sigma) to reduce peritubular cells. Then testicular cells were washed and plated in medium with gentamicin (0.02 g/L; Sigma), maintained in a humidified atmosphere at 34 °C with 5% CO_2_ for 6–8 hrs. Germ cells were harvested by gentle shaking and suction gently^54^. The residual aggregate was consisted of sertoli cells (SC). To collect the spermatogonia (SPG), 7–8 dpp male mice were employed. For the sub-group meiosis prophase I spermatocytes, the method of STA-PUT was used and cells were isolated according to the diameters. leptotene (LS): 8–10 μm, zygotene (ZS): 10–12 μm, and pachytene & diplotene (PDS): 14–18 μm. Spermatids (SPD) were isolated by the method of flow cytometry with the Hoechst 33342 staining. According to a published report^55^, the identification of the testicular cells were showed in Supplemental Figure 8.

### RNA isolation and real-time PCR

Total RNA was extracted from the wild type and *Ndrg3*^+/−^ mouse indicated, tissues or isolated primary cells homogenized in TRIzol reagent (Invitrogen), followed by RNA precipitation. cDNA was synthesized with a reverse transcription kit (TaKaRa). Real-time PCR was performed using SYBR Premier EX Taq (TaKaRa). Genes were amplified with the indicated primers (Supplemental Table). Relative levels of mRNAs were calculated using MX3500pro software and normalized to the levels of endogenous *β-Actin* in the same samples.

### Western blot

Tissue or cell extracts containing 30 μg proteins were resolved by SDS-PAGE and transferred to nitrocellulose (NC) membrane (Millipore Corp). After probing with primary antibodies, the membranes were incubated with Odyssey fluro-800–conjugated anti-rabbit/mouse IgG antibodies (Li-Cor). The primary antibodies used were NDRG3 (Santa Cruz Biotechnology, 1:1000), pERK1/2 (CST, 1:1000), ERK1/2 (CST, 1:1000) and β-Actin (Sigma, 1:10000).

### ChIP-qPCR assay

Germ cells cultured were treated with or without U0126 (15 μM) overnight. Cells were harvested and DNA was precipitated with a polyclonal anti-pCREB antibody, or mouse IgG as a negative control. Precipitated DNA was amplified with the following primer sets for the Rad51 promoter region: P1 5′-ATTCTGGGTTATGTAGTCCT-3′ (upper) and P2 5′-TCCCGCCAAATCCTCACGCT-3′ (lower). The PCR products were confirmed by sequencing.

### Statistical analysis

All statistical data were analyzed with GraphPad Prism version 5. The statistical data of litter sizes, sperm number, testis weight, testosterone levels, number of spermatocytes cells and relative mRNA expression were presented as means ± SEM. ANOVA or Student’s *t* test were used for statistical comparison to determine significance. The difference of the number of spermatogonium, MMI and MMII cells as well as the ratios of MMII to MMI were tested for significance using the Mann-Whitney *U*-test as described previously^32^. Statistical significance set: NS, P > 0.05; *P ≤ 0.05; **P ≤ 0.01. All presented results were from at least 3 independent experiments.

## Additional Information

**How to cite this article:** Pan, H. *et al. Ndrg3* gene regulates DSB repair during meiosis through modulation the ERK signal pathway in the male germ cells. *Sci. Rep.* **7**, 44440; doi: 10.1038/srep44440 (2017).

**Publisher's note:** Springer Nature remains neutral with regard to jurisdictional claims in published maps and institutional affiliations.

## Supplementary Material

Supplemental Figures

## Figures and Tables

**Table 1:** Number of SPG, MMI, and MMII cells referred to 1000 cells at mid-pachytene.

Treatment	Average number per animal			MMII/MMI
	SPG (mean ± SEM)	MMI (mean ± SEM)	MMII (mean ± SEM)	
Wild-type	3.22 ± 0.41	90.00 ± 8.2	194.44 ± 18.45	2.16
Ndrg3^+/−^	4.45 ± 2.48	72.82 ± 6.94^*^	149.73 ± 11.36^*^	2.05

SPG, spermatogonial mitoses; MMI, meiotic metaphase I; MMII, meiotic metaphase II. *P < 0.05; Mann-Whitney U-test.
