# Supplementary material for: Ndrg3 gene regulates DSB repair during meiosis through modulation the ERK signal pathway in the male germ cells
Source: Sci Rep. 2017 Mar 14;7:44440. doi: 10.1038/srep44440 (PMC5349515; doi:10.1038/srep44440)

Ndrg3 gene regulates DSB repair during meiosis through modulation the ERK signal pathway in the male germ cells

Hongjie Pan[a,b], Xuan Zhang[a,b], Hanwei Jiang[c], Xiaohua Jiang[c], Liu Wang[c], Qi Qi[a,b], Yuan Bi[a,b], Jian Wang[a,b], Qinghua Shi[c *], Runsheng Li[a,b *]

a. WHO Collaborating Center for Research in Human Reproduction, Key Laboratory of Contraceptive Drugs and Devices of NPFPC, Shanghai Institute of Planned Parenthood Research, Shanghai, China. 200032
b. Institute of Reproduction and Development, Fudan University, Shanghai, China. 200032
c. Laboratory of Molecular and Cell Genetics, CAS Key Laboratory of Innate Immunity and Chronic Disease, CAS Hefei Institutes of Physical Science, Hefei National Laboratory for Physical Sciences at Microscale, School of Life Sciences, University of Science & Technology of China, Hefei, China. 230027

* To whom correspondence should be addressed

Prof. Runsheng Li,

Key Laboratory of Contraceptive Drugs and Devices of NPFPC, Shanghai Institute of Planned Parenthood Research, Shanghai, China, 200032.

Institute of Reproduction and Development, Fudan University, Shanghai, China

Phone number: 0086-021-64437446

Fax number: 0086-021-64046128.

E-mail: runshengli2007@163.com

Prof. Qinghua Shi
Hefei National Laboratory for Physical Sciences at Microscale, and School of Life Sciences, University of Science and Technology of China, Hefei, Anhui 230027, China
Tel & Fax: +86-551-63600344.
E-mail address: qshi@ustc.edu.cn

Supplementary table

Name		Sequence
Spo11	Sense:	5'-TGACATTTCCTGTATGCTGA-3'
	Anti-sense:	5'-ACATTATCTCGATGCCGTAG-3'
Sycp1	Sense:	5'-AGAACAGTCATCAGCGAAGA-3'
	Anti-sense:	5'-AGGCTCAAGAGATCAGCAGT-3'
Sycp3	Sense:	5'-CCAGAGAATGAAAGCAATCA-3'
	Anti-sense:	5'-CAATCGTGGAGAGAACAACT-3'
Rad50	Sense:	5'-ACTGTTGGGGTACTTTCCTA-3'
	Anti-sense:	5'-TCCGATTCTGTTGACTTGAG-3'
Mre11	Sense:	5'-AGTATTTAGTATCCACGGCA-3'
	Anti-sense:	5'-GGGCTGAGACACATAGAAGA-3'
Rad51	Sense:	5'-GGTTACCATACAGTGGAGGC-3'
	Anti-sense:	5'-TGCTACATTATCTAGGACATCG-3'
Dmc1	Sense:	5'-ATCCAGGAGCAACTATGACC-3'
	Anti-sense:	5'-AATAAGCAGCAAGAAGCAGT-3'
Fzr1	Sense:	5'-GACCAGGACTATGAGCGAAG-3'
	Anti-sense:	5'-CTGACTTTTGTTGCTAACGG-3'
Brca1	Sense:	5'-AAATGGATTTATCTGCCGTC-3'
	Anti-sense:	5'-TGTGGTCACACTTTGTGGAA-3'
Brca2	Sense:	5'-GGGTTTATCAAGGGATGTTA-3'
	Anti-sense:	5'-AGCAAGACCTATCGGTTTTA-3'
Gfra1	Sense:	5'-TCATTGGCAGAAACATCGTA-3'
	Anti-sense:	5'-TCATCCATCCACACTAGGCT-3'
Uchl1	Sense:	5'-TTCTGTTCAACAACGTGGAC-3'
	Anti-sense:	5'-AGAGACCGTAGAGAGCAAGG-3'
Rec8	Sense:	5'-GTGAAGCGTGAATACCTAAA-3'
	Anti-sense:	5'-GGCACTGTTGAAAGTAGACC-3'
Hsp70-2	Sense:	5'-CGCCTCACCCAACTAGATAT-3'
	Anti-sense:	5'-TCATATCGGACTGCACTGTG-3'

Supplementary Figure 1. No apparent hormone defect was detected in the *Ndrg3*$^{+/-}$ mice compared with the wild type mice. The testosterone hormone level of the wild type and the *Ndrg3*$^{+/-}$ mice was comparable, indicating that the meiosis defect was mainly due to the development failure of the male germ cells. N=8, P=0.197.

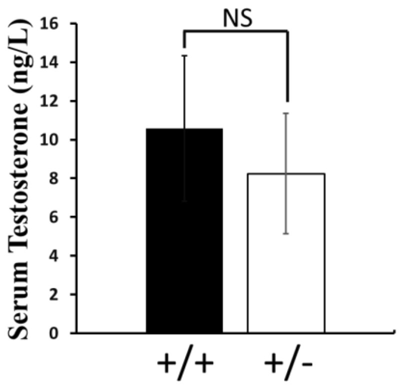

Supplementary Figure 2. Germ cells at different stage of meiosis in testes stained by Giemsa. a, mid-pachytene nuclei with linear chromatin and scattered dark-stained granules were showed. Note the dark stained sex vesicles locating in the periphery of nuclei (black dashed circles). **b,** Metaphase stage of spermatogonial (M) with n=40. **c**, Metaphase I (M I) with 19 autosomal bivalents and end-to-end associated X, Y chromosome (red dashed circle). **d**, Metaphase II (M II) with n=20.

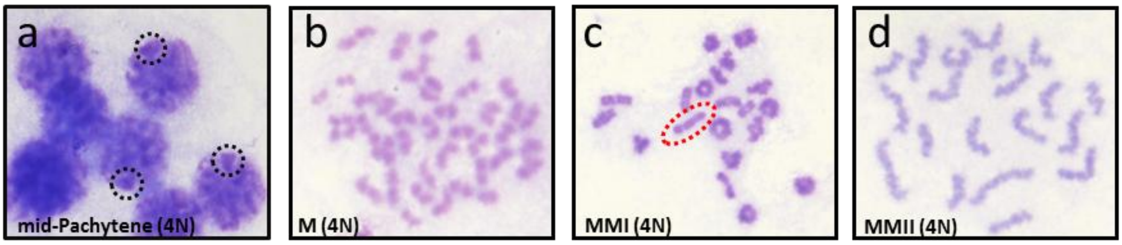

Supplementary Figure 3. No apparent defects were detected in the homologous chromosomes synapsis and crossover between the *Ndrg3*$^{+/-}$ and wild type testes. Double immunofluorescence of surface-spread chromatin preparations were performed with the wild type and *Ndrg3*$^{+/-}$ testes. (a), SYCP3 (red), SYCP1 (green). (b), SYCP3 (red), MLH1 (green). (c), Quantification of the MLH1 foci. Note: the PAR region was not included in the quantification.

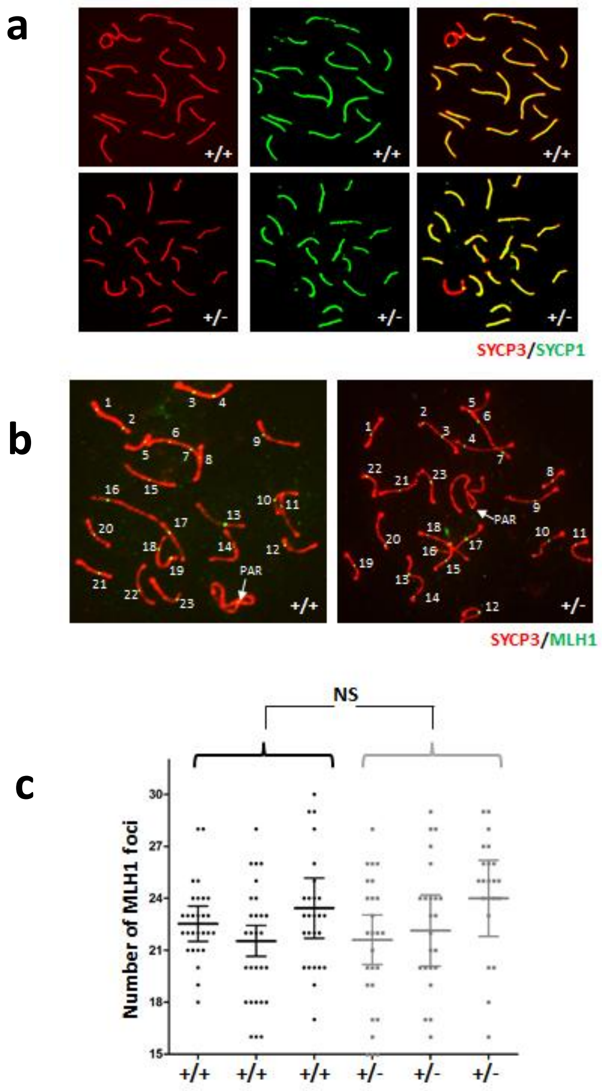

Supplementary Figure 4. The inactivation of ERK signaling was confirmed by western blot assay. Abbreviation: U0, U0126 (0 μM). U15, U0126 (15 μM). U30, U0126 (30 μM)

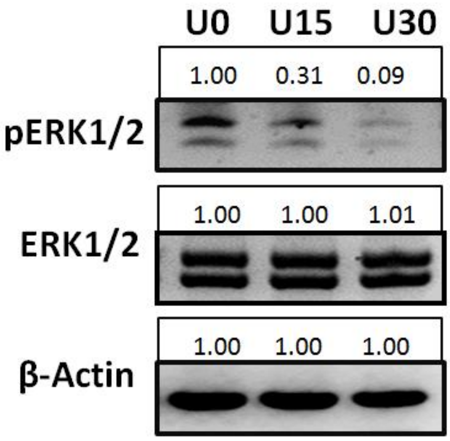

Supplementary Figure 5. Western blot assay of the indicated proteins in the cultured wild type and *Ndrg3*$^{+/-}$ male germ cells.

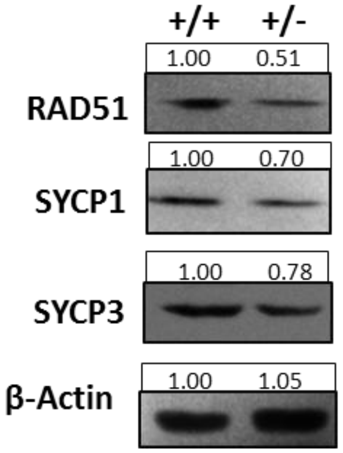

Supplementary Figure 6. No apparent difference in the number of the pups between the *Ndrg3*$^{+/-}$ and wild type mice. The mean number of pups born to mated *Ndrg3*$^{+/-}$ (+/-) and the wild type (+/+) in the age of 10-12 weeks old.

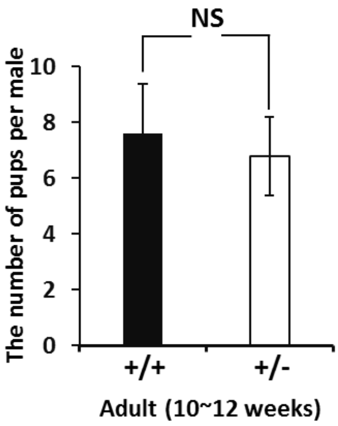

Supplementary Figure 7. Real-time assay showed the mRNA level of *Ndrg1* in mouse testis at the indicated time points.

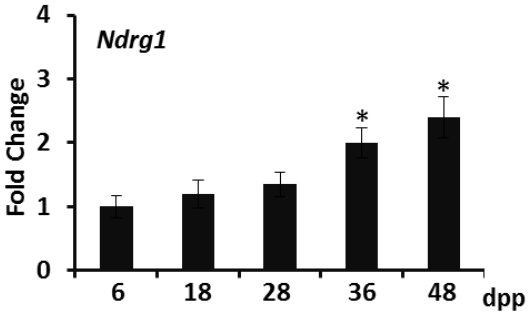

Supplementary Figure 8. Identification of the isolated testicular cells. a, The identification of spermatogonia (SPG), spermatids (SPD), and sertoli cells (SC) was performed by real-time PCR assay. The sub-groups of the meiotic prophase I spermatocytes (leptotene (LS), zygotene (ZS), pachytene & diplotene (PDS)) were discriminated according to the morphology characteristics of SYCP3 protein. Note: Because of the similar diameters, pachytene and diplotene spermatocytes could not be discriminated.

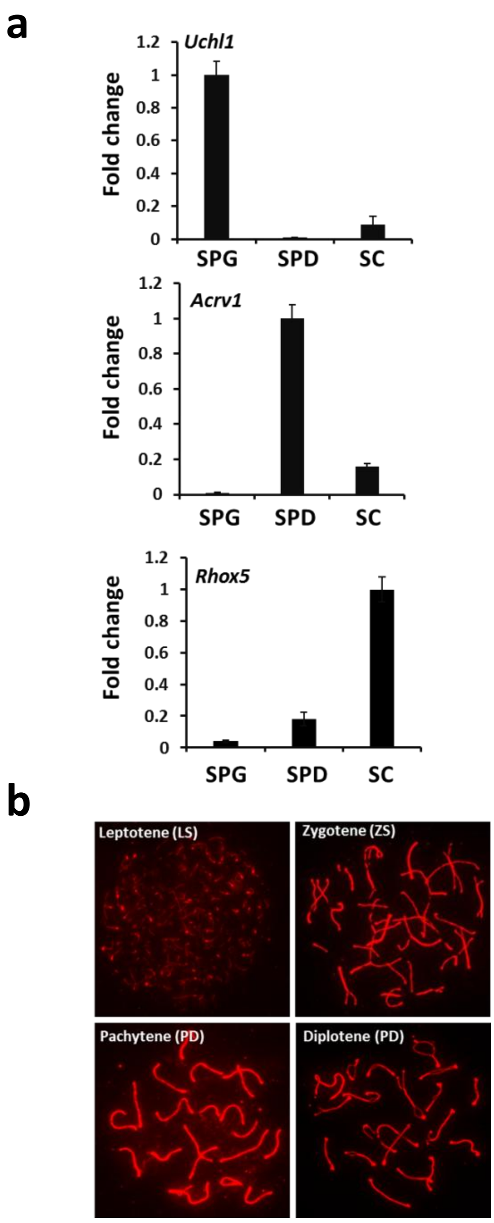

Supplementary Figure 9. Western blot assay showed the expression pattern of NDRG3 in testes of mice at the indicated time points (a); The expression of NDRG3 in spermatogonia, spermatocytes of different sub-stages in meiosis prophase I and sertoli cells (b).

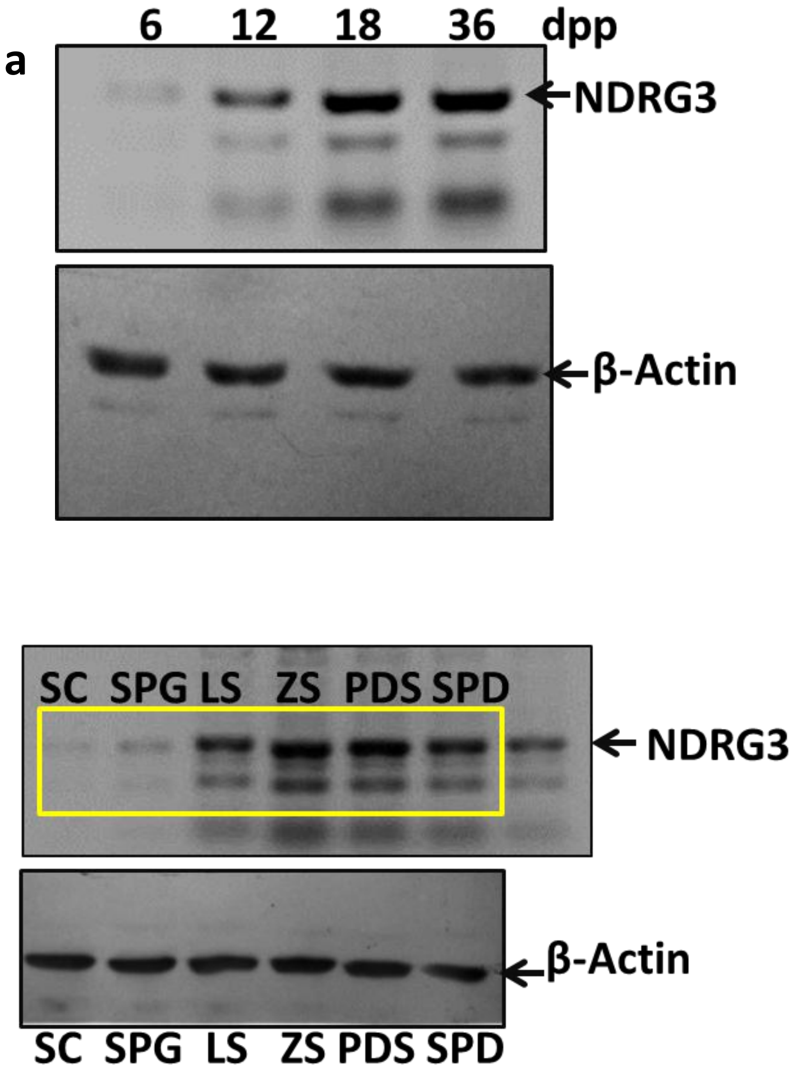

Supplementary Figure 10. Agarose gel photograph of Surveyor Nuclease Assay demonstrated digestion products of the predicted size from pup #2, #3, #5, #7, #8 and the WT pup #1, #4, #6, #9, #10, #11 (a). Western blot assay showed the level of the NDRG3 protein in the wild type and heterozygous testes of mice (b).

a

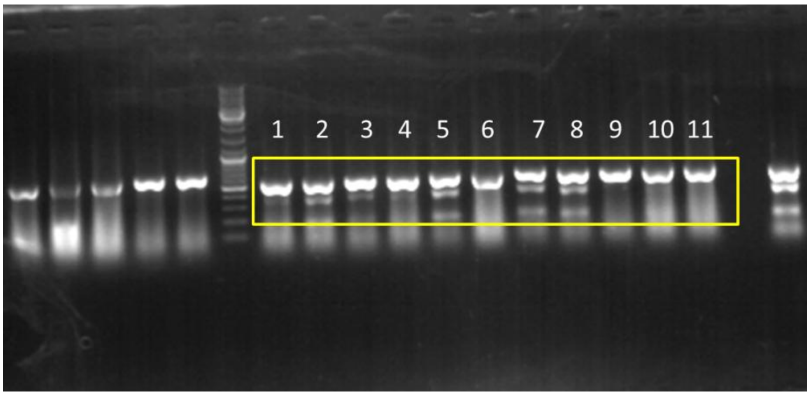

b

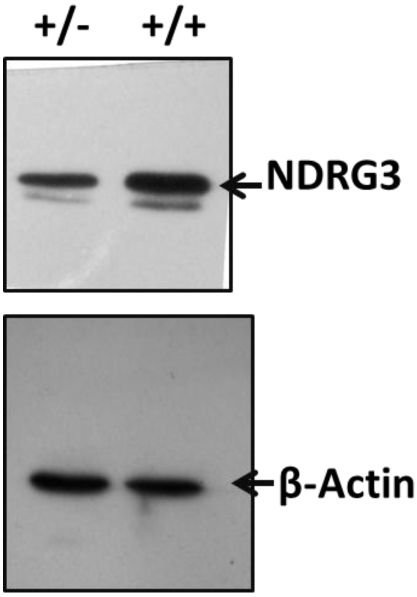

Supplementary Figure 11. Western blot assay showed the indicated protein levels between the wild type and *Ndrg3*$^{+/-}$ testes.

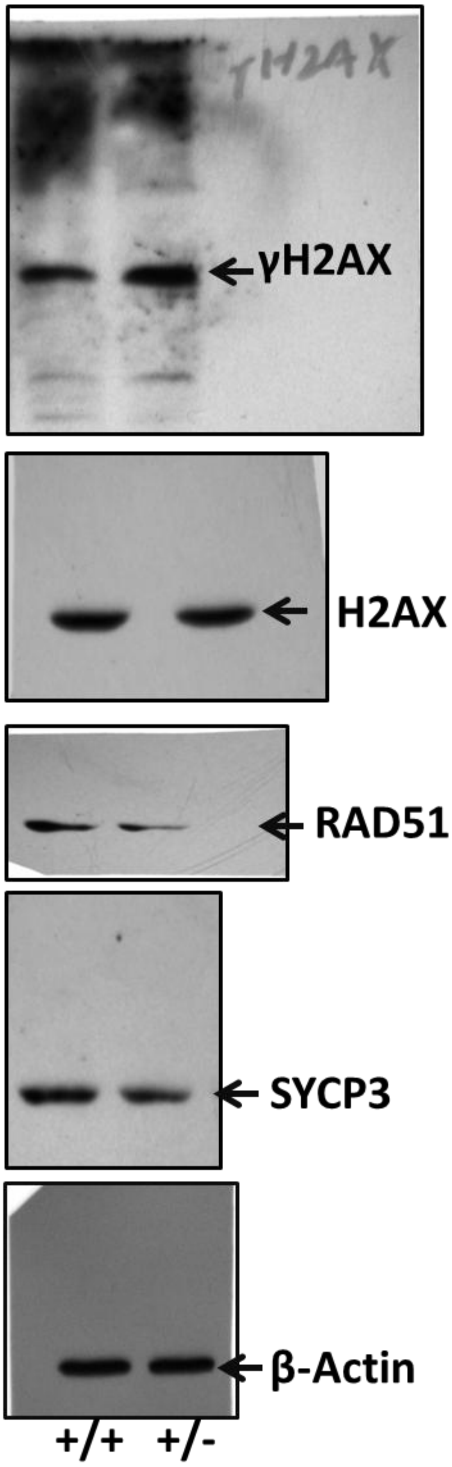

Supplementary Figure 12. Western blots showed the indicated protein levels. The wild type male germ cells were isolated and cultured with or without lactate (15mM) or U0126 (15μM) respectively. The pERK1/2, ERK1/2, and beta-Actin levels were determined (a). The pERK1/2, ERK1/2, NDRG3 and beta-Actin levels were determined with (15 mM，30 mM) or without the treatment of lactate (0 mM) in the cultured primary male germ cells (b). The expression of RAD51 in the isolated and cultured wild type and *Ndrg3*$^{+/-}$ male germ cells (c). The ERK1/2 signal in the wild type and *Ndrg3*$^{+/-}$ testes was determined (d).

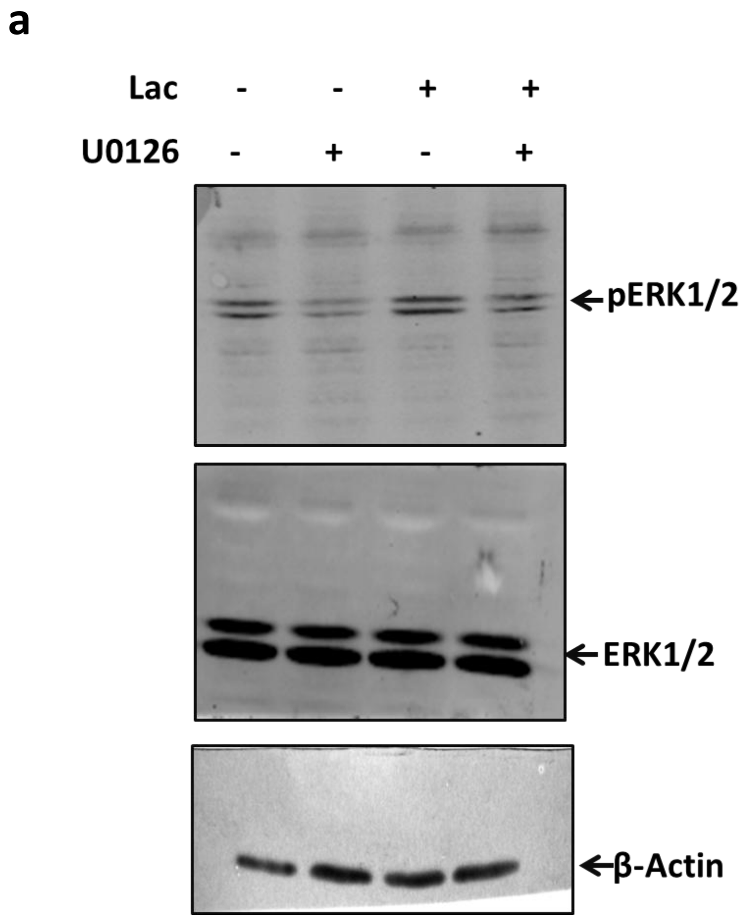

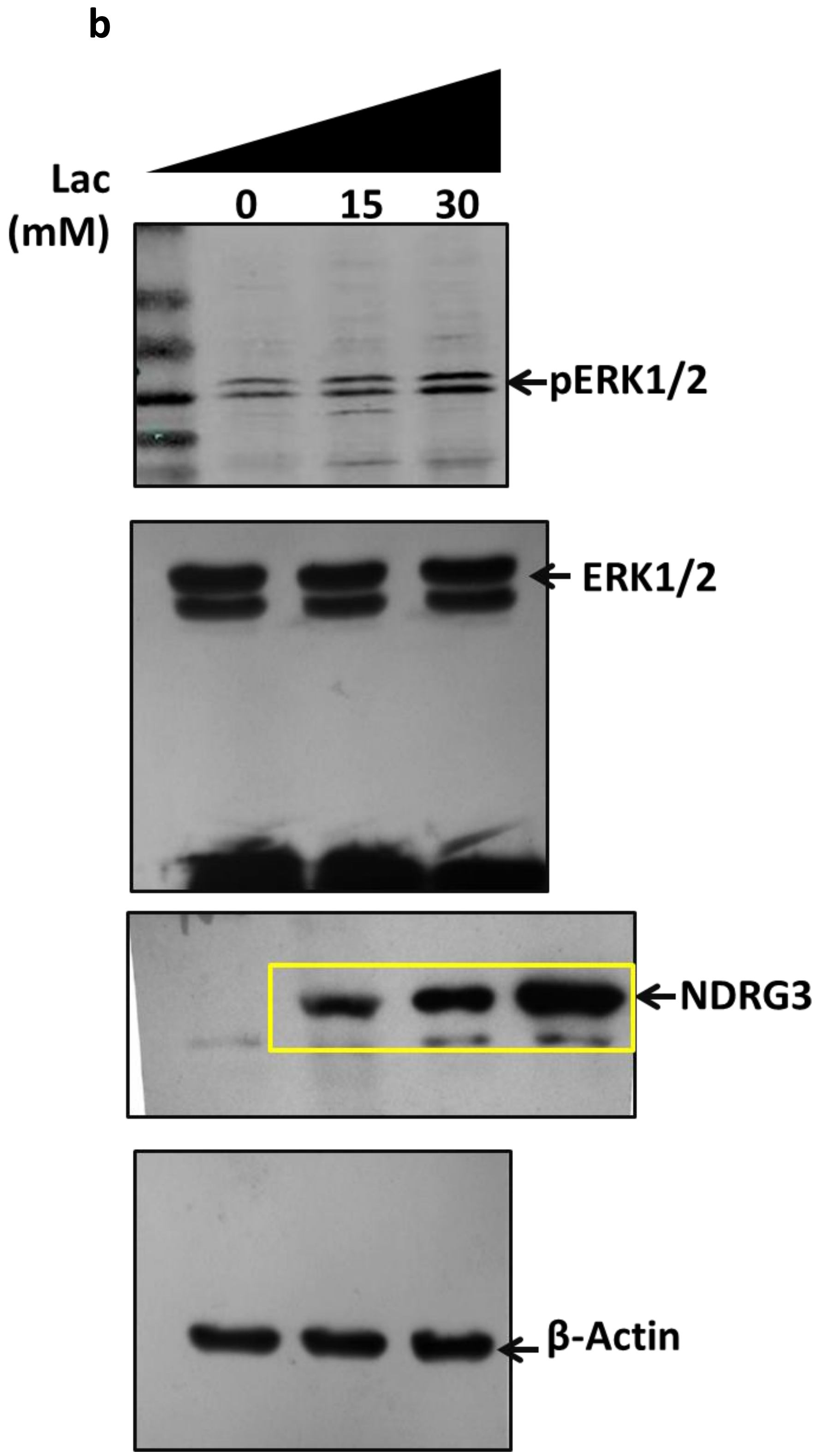
b
Lac
(mM)
0
15
30
pERK1/2
ERK1/2
NDRG3
β-Actin

c

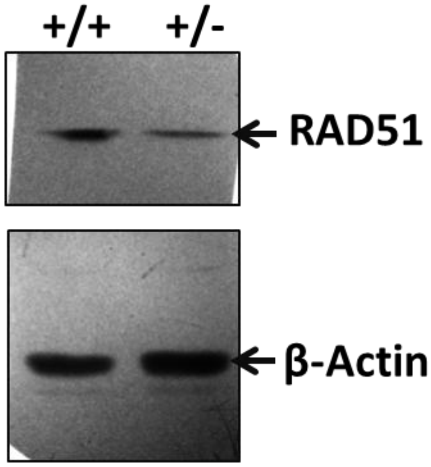

d

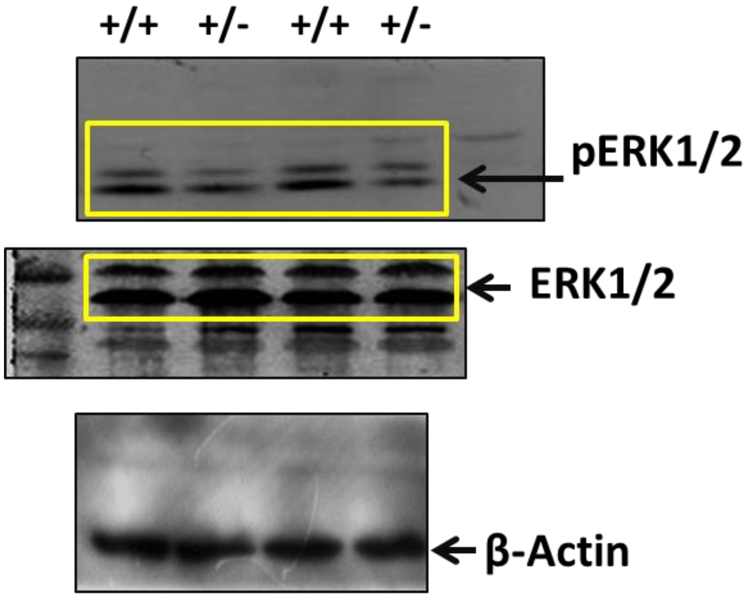

Supplementary Figure 13. The activation of ERK-CREB signaling was determined by western blot assay. The wild type and *Ndrg3*$^{+/-}$ male germ cells were isolated and cultured with or without lactate. The pERK1/2, ERK1/2, pCREB, CREB and β-Actin were determined by specific antibodies.

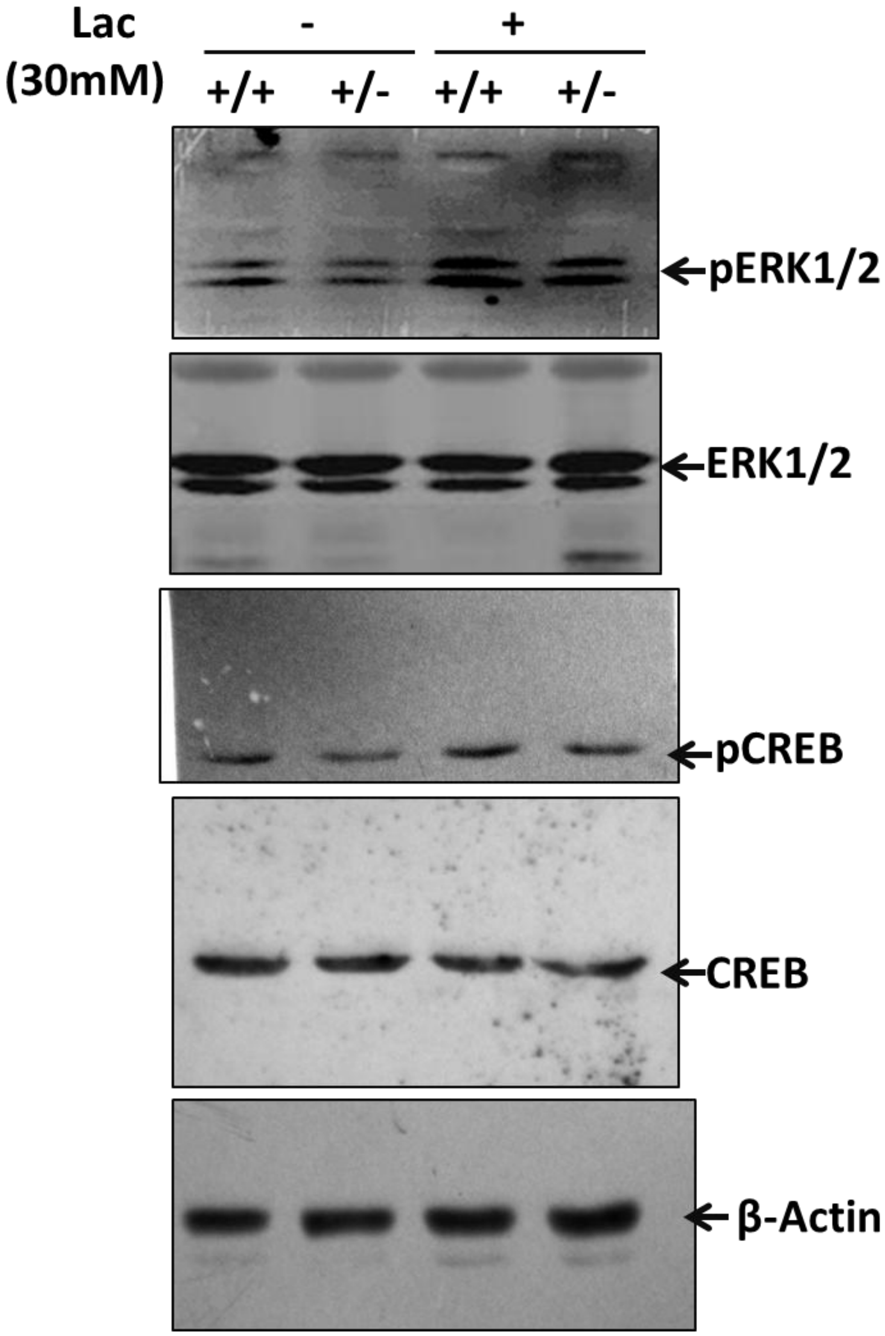

Supplement: Supplemental Figures [file srep44440-s1.pdf]
